# Genetic analysis and clinical significance of a rare t(1;12)(q21;p13) in a patient with high‐risk myelodysplastic syndrome

**DOI:** 10.1002/mgg3.1893

**Published:** 2022-02-22

**Authors:** Fang Fang, Ru Jia, Congyan Liu, Hong Zhao, Wanling Sun

**Affiliations:** ^1^ Department of Hematology, Xuanwu Hospital Capital Medical University Beijing China

**Keywords:** chromosome translocation, fluorescence in‐situ hybridization, genetic analysis, karyotype

## Abstract

To explore the genetic and clinical features of a rare t(1;12)(q21;p13) in a patient with myelodysplastic syndrome (MDS). A 53‐year‐old male was diagnosed as high‐risk MDS, and died in a short period. A complete cytogenetic analysis of bone marrow by conventional G‐banding karyotyping was performed at the time of initial evaluation. On the basis of chromosome karyotype, interphase and metaphase fluorescence in‐situ hybridization (FISH) were carried out to further confirm the abnormal karyotypes. Reverse‐transcription polymerase chain reaction (RT‐PCR) was performed to determine *ETV6/ARNT* fusion gene status. G‐banding revealed karyotype 47, XY, +8, der(12) t(1;12)(q21;p13). FISH with the centromere 8 probe verified the trisomy 8, and the *ETV 6* break‐apart probe suggested heterozygous loss of *ETV6* allele located in short arm of chromosome 12. Subsequently, the painting probe of whole chromosome 12 further confirmed the part break of short arm of chromosome 12, and the 1q21/1p36 probe yielded three signals of 1q21 and two signals of 1p36. The results of FISH were in accordance with the karyotype completely. No *ETV6/ARNT* fusion gene was detected by PCR. T(1;12)(q21;p13) is a rare abnormal karyotype, and the limited reports cannot supply definite clinical significance. Rapid deterioration of our case suggests this translocation of chromosome might have a poor effect on the survival of MDS.

## Graphical abstract

t(1;12) (q21;p13) is rarely observed and has only been reported in hematologic disease. We identify the locations of chromosome breaks, check the fusion gene, and summary clinical reports.

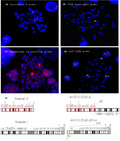

## 1 INTRODUCTION

Advances in cytogenetic and molecular genetics have widened the recognition of hematologic malignancy. Chromosome abnormalities often influence related gene expression. Translocation and inversion of chromosomes can result in a split gene and form a fusion gene, which may be translated into a fusion protein and disrupt normal function. Today, many genes involved in abnormal karyotypes have been located, cloned, and sequenced, and the functions of relevant fusion proteins have been identified (Erickson et al., 1992; Gao et al., 1991). The considerable progress in genetics has provided an important basis for elucidating the molecular mechanisms in the pathogenesis of several hematological neoplasms (Al‐Harbi et al., 2020; Peterson & Zhang, 2004). Some recurrent cytogenetic abnormalities have already applied critical evidence for the diagnosis and targeted therapy in certain disorders, for example, tyrosine kinase inhibitor therapy in chronic myeloid leukemia (CML) with t(9,22)(q34;q11) (Jabbour & Kantarjian, 2018) and all‐trans retinoid acid therapy in acute promyelocytic leukemia with t(15,17)(q22;q21) (Wang & Chen, 2008). However, the clinical significance of other chromosome abnormalities still requires clarification.

This paper reports a patient carrying a rare karyotypic abnormality including the reciprocal translocation of chromosomes 1 and 12, t(1;12) (q21;p13). This translocation is rarely observed and has only been reported in hematologic diseases. Therefore, the cytogenetic and molecular genetics of the patient were further analyzed.

## 2 PATIENT AND METHOD

### 2.1 Patient information

**FIGURE 1:**
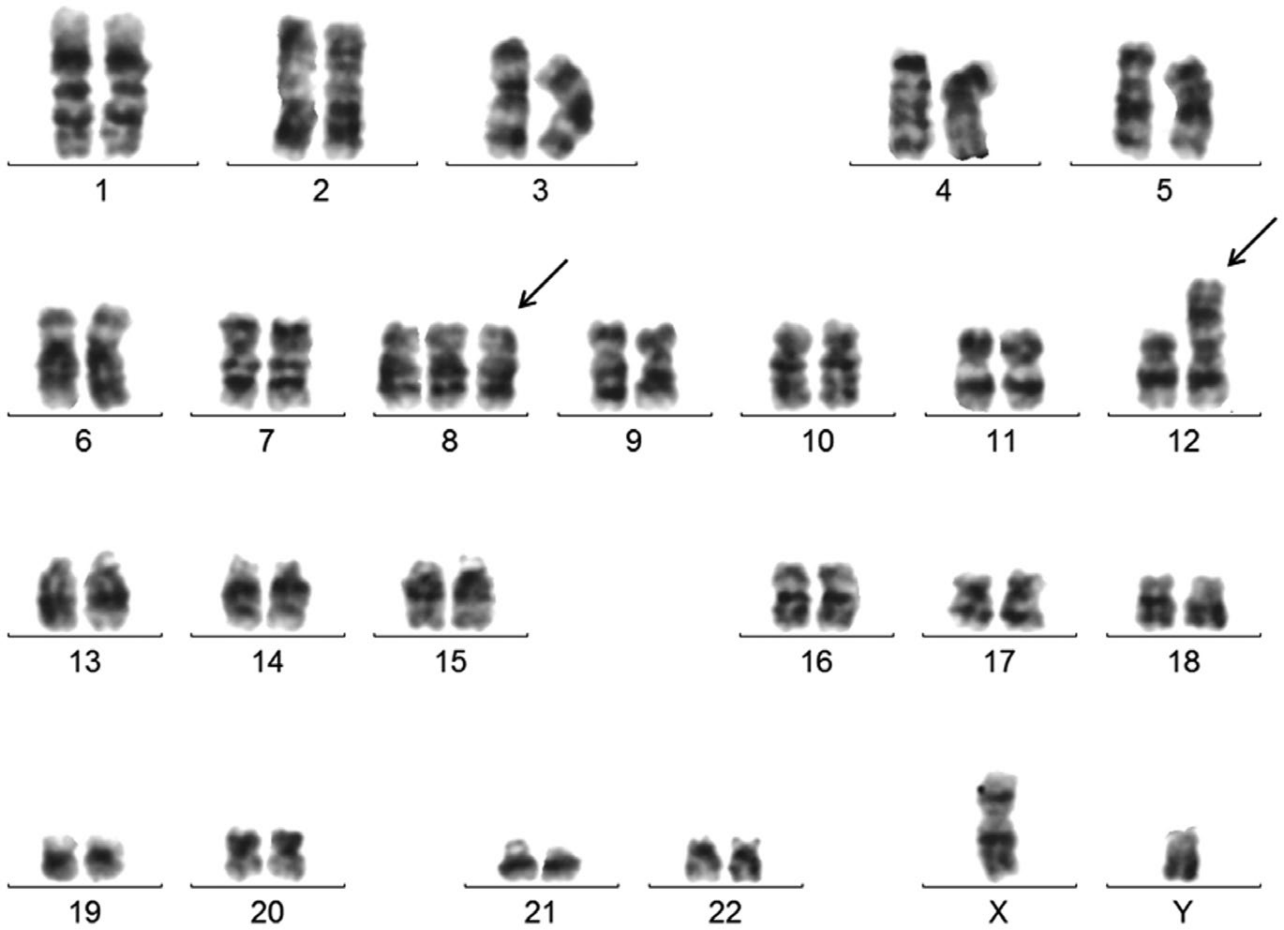
Karyotype analysis based on G‐banding in the male patient: 47, XY, +8, der(12)t(1;12)(q21;p13) [10]

A 53‐year‐old male was admitted to the hospital with a history of fatigue, poor appetite accompanied by weight loss for 6 months, and hoarseness with dyspnea for 2 months. Physical examination showed obvious pallor. The complete blood count was leukocytes 3.28 × 10^9^/L (including 5% blasts), hemoglobin 45 g/L, and platelets 57 × 10^9^/L. Bone marrow aspiration smear showed hypercellularity and dysplasia in granulocytic cells and megakaryocytes, 6% blast and 19% ring sideroblasts. Immunophenotyping by flow cytometry revealed that blast cells were positive for CD 13, CD34, CD38, CD117, and HLA‐DR. No RUNX1‐RUNX1T1, CBFB‐MYH11, or PML‐RARA fusion genes and no mutations of FLT3‐ITD, CEBPA, or NPM1 were detected. Conventional cytogenetic analysis of bone marrow using a G‐banding technique revealed a karyotype of 47, XY, +8, der(12)t(1;12)(q21; p13) in available 10 metaphases (Figure 1).

The arterial blood gas analysis indicated pH 7.411, PaO2 58.4 mmHg, SaO2 90.3%, and PaCO2 41.7 mmHg. Laryngoscopy showed incomplete paralysis of the vocal cords, with normal appearance. PET‐CT scan, performed in another hospital, showed an abnormal radioactivity concentration between the right thyroid gland and cricoid cartilage. Biopsy of the vocal cords was recommended, but the patient refused the high‐risk operation. Therefore, a diagnosis of myelodysplastic syndrome with excess blast‐2, with ring sideroblasts (MDS‐EB‐2‐RS), vocal cord incomplete paralysis, and type I respiratory failure was established. Decitabine 50 mg/d for 3 days were administrated, supplemented with erythropoietin and other supportive treatment. Another course of decitabine was given 1 month later and the patient was discharged home. One month later, the patient visited an emergency department and died of unknown cause in a short time, and pulmonary infection was highly suspected.

### 2.2 Cytogenetic analysis

Cytogenetic analysis using standard G‐banding techniques on heparinized BM samples was performed as described previously (Bates, 2011). Chromosome identification and karyotype description used the International System for Human Cytogenetic Nomenclature (ISCN) (McGowan‐Jordan et al., 2016).

### 2.3 Fluorescence in situ hybridization (FISH)

On the basis of chromosome karyotype, interphase and metaphase FISH were carried out to further confirm the abnormal karyotypes following the standard protocols (Kearney et al., 2002). Briefly, the common MDS FISH panel including CEPX/CEPY, CEP8, 5q33‐34/5p15.2, 5q31/5p15, 7q31(D7S486)/CEP7, 7q31(D7S522)/CEP7, 7q31 (D7S522)/CEP7, 20q12, and ETV6 break‐apart probe (Abbott) were performed first, then the whole chromosome 12 painting probe (Kreatech Diagnostics) and 1q21/1p36 probe (GPMEDICAL) were hybridized subsequently.

### 2.4 Reverse‐transcription polymerase chain reaction (RT‐PCR)

RNA was extracted (Thermo Fisher Scientific) and reverse transcription (Promega) were operated according to the manufacturer's instructions, and the product was used for the polymerase chain reaction (PCR) analyses of *ETV6/ARNT* fusion gene. The synthesized PCR products were electrophoresed onto a 2% agarose gel and stained with ethidium bromide. The primer designed for *GAPDH* (OMIM18400), *ETV6* (OMIM126110), and *ARNT* (OMIM 600618) as follow.

*GAPDH* (NM 001289746.1). Forward primer 5′‐ATCGCTCAGACACCATGGGGAAG‐3′. Reverse primer 5′‐CAAAGTTGTCATGGATGACC‐3′.

*ETV‐6* (NM 001987.4).Forward primer (primer 1) 5′‐CTTGCAGCCAATTTACTGG‐3′. Reverse primer (primer2) 5′‐AGAGGGTAGGACTCCTGGTG‐3′.

*ARNT* (NM 001286036.1). Forward primer (primer3) 5′‐CAGAGCTCTGCTCATCATCCGA‐3′. Reverse primer (primer4) 5′‐CATGGCGGCGACTACTGCCAAC‐3′.

## 3 RESULTS

### 3.1 FISH confirmation of the karyotype

**FIGURE 2:**
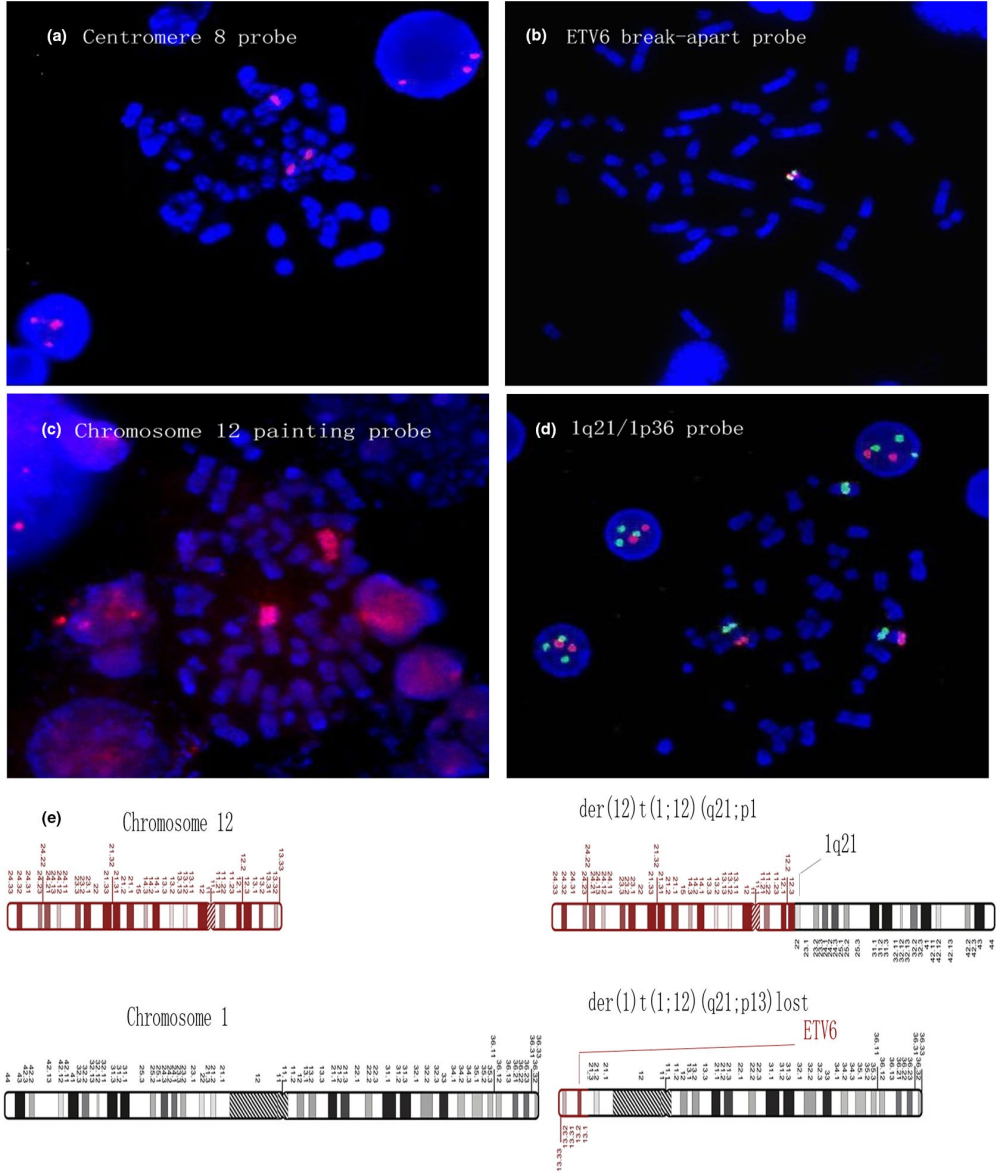
FISH image of the bone marrow (a) chromosome 8 centromere probe: three red signals. (b) *ETV6* break‐apart probe: One fusion signal. (c) Whole chromosome 12 painting probes: red signals on one complete chromatid and part of the chromosome12 covering the centromere. (d) 1q21 (green)/1p36 (red) probe: two red signals and three green signals. (e) Schematic map of t(1;12)(q21;p31)

The first line FISH revealed the trisomy 8 with three red signals of the centromere 8 probe (Figure 2a), and the heterozygous loss of *ETV6* allele located in the short arm of chromosome 12 with one fusion signal of the *ETV6* break‐apart probe on the normal chromosome 12 (Figure 2b). At the same time, there were no ‐X/‐Y, del(5q)/−5, del(7q)/−7, or 20q‐ found by FISH. These results verified +8 and the translocation of 12p13 with a breakpoint centromeric to the *ETV6* allele, while the derivative chromosome carrying the broken 12p with *ETV6* allele had been lost.

Furthermore, to verify the karyotype of t(1,12)(q21;p13), the metaphase FISH using a painting probe of whole chromosome 12 labeled by red fluorescence was further carried out. In metaphases, one chromosome 12 was painted completely red and another was partly labeled red, including the centromere. This further confirmed the break of short arm of one chromosome 12 (Figure 2c). The banding of the unlabeled part of the derivative chromosome 12 was consistent with the telomeric part of 1q in DAPI staining, supporting the partner chromosome of this translocation is chromosome 1.

On the basis of G‐banding, there were two normal chromosome 1 and chromosome telomeric to 1q21 was translocated to the derivative chromosome 12. Therefore, 1q21/1p36 probe was chose to evaluate chromosome 1. The hybridization yielded three signals of the 1q21 (green signals) and two signals of 1p36 (red signals), both in metaphases and interphases (Figure 2d). Especially, the metaphases clearly showed two normal chromosome 1 and the derivative chromosome 12 carrying 1q21. Thus, the FISH results were in accordance with the karyotype 47, XY, +8, der(12) t(1;12)(q21;p13) completely.

### 3.2 RT‐PCR detecting ETV6/ARNT fusion gene

According to the previous FISH results, the breakpoint of t(1;12)(q21;p13) in chromosome 12 centromeric to *ETV6* allele. As a result, *ETV6* was not involved in the translocation. To confirm this inference, RT‐PCR was conducted to detect *ETV6/ARNT* fusion gene, which had been reported the result of t(1;12)(q21;p13) (Otsubo et al., 2010; Salomon‐Nguyen et al., 2000). In our case, the RT‐PCR was negative and there was no *ETV6/ARNT* fusion gene detected. This result was in accordance with our expectation.

## 4 DISCUSSION

Generally, patients with MDS exhibit genetic abnormalities, and some of such abnormalities represent independent prognostic variables in MDS. The patient we reported was diagnosed as MDS‐EB2, with a 47, XY, +8, der(12)t(1;12)(q21;p13). Trisomy 8 is a relative common abnormality in various hematological diseases, which is neutral for the prognosis in MDS. While the t(1;12)(q21;p13) is rarely reported.

A retrospective review of literature revealed totally six cases carrying t(1;12)(q21;p13), all of which were associated with hematologic disease (Table 1). The first case with t(1;12)(q21;p13) was reported in 1984 (Lewis & MacKenzie, 1984), and the diagnosis was multiple myeloma. Then, another five cases were reported in succession, including one acute myeloid leukemia (AML) (Salomon‐Nguyen et al., 2000), one high‐risk myelodysplastic syndrome (Sánchez et al., 2000), one CML (Palandri et al., 2009), and two acute lymphoblastic leukemia (ALL) (Heerema et al., 2004; Otsubo et al., 2010). Among the six cases, three were three male, and three female. The ages ranged from 2 to 66 years. The MDS case exhibited only t(1;12) (q21;p13); the other five cases had one to three additional abnormalities. Among the six reported cases, molecular biological study of the bone marrow was performed in three cases (Otsubo et al., 2010; Salomon‐Nguyen et al., 2000; Sánchez et al., 2000). The fusion gene *ETV‐6/ARNT* was detected in two cases, and even the related fusion protein was verified in the AML patient (Salomon‐Nguyen et al., 2000). We here reported the seventh patient with t(1,12) (q21;p13) and confirmed this rare finding by FISH.

**TABLE 1:** Clinical characterization of t(1;12)(q21;p13) in patients

Year	Sex	Age	Diagnosis	Karyotype	Gene	Protein	Survival time
1984	F	66	MM IgA κ	45, XX, −13, del(2)(q21), t(1;12)(q21;p13), 14q+	—	—	10 m
2000	M	5	AML‐M2	47, XY, t(1;12)(q21;p13), +21/46, XY	ETV6/ARNT	TEL‐ARNT and ARNT‐TEL	—
2000	M	64	MDS‐RAEB‐t	46, XY, t(1;12)(q21;p13)	No ETV6 involved	—	—
2004	F	—	ALL	46, XX, t(1;12)(q21;p13), del(7)(q22q36), t(12;18)(p13;q21)	—	—	—
2009	F	—	CML	46, XX, t(9;22)(q34;q11), t(1;12)(q21;p13)	—	—	47 m
2010	M	2	T‐ALL	46, XY, t(1;12)(q21;p13),‐16, +r[cp11]/46, XY	ETV6/ARNT	—	—
Our case	M	55	MDS‐RAEB2‐RS	47, XY, +8, der(12)t(1;12)(q21;p13)	No ETV‐6 involved	—	2 m

Translocation resulting from the break apart of 12p13 usually involves *ETV6* gene in hematologic malignancies, such as t (5;12) (q31‐33; p13) in chronic myelomonocytic leukemia (Apperley et al., 2002; Di Giacomo et al., 2021) and t (12;21) (p13;q22) in ALL (Montaño et al., 2020; Shurtleff et al., 1995). The aryl hydrocarbon receptor nuclear translocator (*ARNT*) gene, also named *HIF‐1β*, locates in 1q21, which play a critical role in driving tumor growth and metastasis (Lee et al., 2021). It has been reported as a partner gene of fusion gene in two cases with t(1,12)(q21;p13)(Otsubo et al., 2010; Salomon‐Nguyen et al., 2000). Therefore, we also tried to detect this fusion gene in our case. The reported breakpoint of the *ETV6* gene was located between exons 4 and 5 or between exons 3 and exon 4, and the *ARNT* gene was located between exons 1 and 2, suggesting these sites were fragile on the chromosomes. Therefore, primers for *ETV6* were designed to target exon 3 (primer 1) and exon 5 (primer 2), and primers for *ARNT* were designed to target exon 1 (primer 3) and exon 2 (primer 4). Under this design scheme, RT‐PCRs were developed and had no positive results, supporting the preceding judgment that the breakpoint of chromosome 12 centromeric to *ETV6* allele and *ETV6* was not involved in this translocation.

Survival information was mentioned for only two previously reported cases (Table 1), so even a preliminary judgment about survival with t(1;12)(q21;p13) is difficult. However, all seven cases, including ours, were malignant hematologic disease, suggesting that t(1;12)(q21;p13) translocation might be a poor prognostic factor. The vocal cord paralysis and dyspnea in our patient, is a rare symptom in patients with MDS. PET‐CT displayed an abnormal radioactivity concentration between thyroid gland and cricoid cartilage, which might be responsible for vocal cord paralysis. It is hardly to exclude the possibility of abnormal myeloid blasts infiltration, without biopsy. Anyway, the patient died in a short period less than 3 months, suggesting the poor prognosis of t(1;12)(q21;p13) in MDS.

## 5 CONCLUSION

To date, t(1;12) (q21;p13) is an uncommon karyotype and may produce *ETV‐6/ARNT* fusion genes and related proteins, but this was not observed in the case reported here. Moreover, t(1;12) (q21;p13) may be associated with a poor prognosis, but its rarity and the limited clinical data make it hard to clarify the mechanism and clinical significance. More cases need to be accumulated.

### ETHICAL COMPLIANCE

All procedures were in accordance with the ethical standards of Xuanwu Hospital Capital Medical University Ethics committee. Informed consent form was obtained from the patient's family.

## CONFLICT OF INTEREST

The authors declare no competing interests.

## AUTHOR CONTRIBUTIONS

W.S. designed the study. F.F., R.J., and H.Z. collected the data. C.L. and R.J. performed the experiment. F.F. R.J., and W.S. interpreted the data. F.F. and W.S. wrote and revised the manuscript.

## Data Availability

Data are available upon reasonable request.
